# Outlines of Pathology

**Published:** 1833-10-01

**Authors:** 


OUTLINES OF PATHOLOGY. By *William Pulteney Alison*, M.D. F.R.S.E Fellow of the Royal College of Physicians, and Professor of the Institute of Medicine in the University of Edinburgh.

THAT department of medicine which treats of the classification, causes, symptoms, and signs of disease, has been called pathology. This is a definition which narrows its limits, for every, the slightest deviation from health to disease is included under the word pathology, just as every thing which has reference to the healthy state is the subject of physiology. There is no branch of medicine so full of interest to the physician as pathology. It is, as it were, the arena upon which the other departments of the science—anatomy, physiology, botany, materia medica, chemistry, are brought into action. Its limits, like those of many of the other sciences, are unbounded; each day extends its domain in the hands of the scientific enquirer, without ever being able to grasp the various modifications of disease which the different textures of an organ will put on. We are aware that there are many in the profession, who think that too much attention has been paid to pathology, and that the study of disease has been sacrificed to the mania of hunting after the prismatic hues of morbid textures; this is an objection which could arise only from the limited sense in which the word pathology has been used by this class, having reference, in their opinion, *only* to appearances after death. But, happily for the science and the valetudinarian world, these are opinions peculiar to a very small portion of the profession. The great competition, and the better order of intellect which are now brought to the study of this subject, aided by the valuable works of our indefatigable neighbours, the French, together with our own native talent, render it unnecessary for us to refute the absurd objections which are advanced against the cultivation of this particular branch of medical science.

Pathology has been divided into many classes; but the most important for our consideration is that into general and special. General pathology means an abstract study of disease, whilst special pathology is confined to those diseases which have a peculiar characteristic feature, which distinguishes them from all other diseases. This division has advantages over the other divisions, into internal and external, either of which may be cultivated independently of the other; whilst general and special pathology are so intimately connected, that the study of the one necessarily involves the study of the other.

Objections may be urged against this division into general and special, we are well aware, as tending to prolong the study of disease. Perhaps, to the practitioner, special pathology is more useful, which affords him a clear knowledge of particular diseases, but, as we have already observed, this cannot exist without implying general pathology. There are certain forms which all diseases have in common; but in particular diseases there are certain distinguishing traits, which are the immediate object of special pathology. General pathology, too, has its disadvantages, for it takes but an abstract view of disease, and from abstraction to error there is but a step.

The first thing for consideration in the study of disease, is a definition, next an exposition of symptoms and causes, then the diagnosis and prognosis, and, lastly, the mode of cure. There is another very important point to observe in the study of general pathology—to pursue that course which is conformable to the study of particular diseases; the mind thus acquires a certain order, by which it arranges its thoughts and classes its several degrees of knowledge.

The abundant supply of standard works on pathology which is daily issuing from the press, both in this country and the Continent, has, at least, removed the charge of neglect with which the profession treated this subject, and affords a reasonable hope of advancing this particular branch to a degree of certainty, which the older writers despaired of attaining. To the work before us, which professes to be merely "Outlines of Pathology," perhaps we should be disposed to pay less attention than it merits, were it not that the recollection of Dr. Alison's lectures left upon our mind an impression, that if he ever came before the public as an author, he would be well received. In this opinion we are not singular.

The objects which he has in view in this book are—"first to state the facts which appear to be ascertained, and the inferences which appear to be fairly deducible from them, in regard to the functions of the living human body in health and disease; and, secondly, to arrange these facts as systematically as possible, in the order in which the functions, as existing in the living body in the adult state, are dependent on one another."

As all the other sciences advance, we should expect that medicine, too, should move forward, if not pari passu, at least not longo intervallo; its steps are indeed few and far between, a circumstance which may be ascribed to the indifference shewn to fixed principles, and their importance in founding a rational system of medicine. Some will assert that medicine is incapable of ever attaining a systematic form, so long as there shall remain any facts unexplained. Though we shall readily admit that there are many facts inexplicable in the present state of the science, yet, as the author justly observes—"if we can follow an order of ascertained dependance of functions on one another, we are entitled to treat the subject systematically or synthetically."

In the prosecution of medicine as a science, there is one great object to be kept in view—the laws which regulate the movements of the living machine must be contra-distinguished from those of art, and are referable alone to vitality. Cullen adopted this view to a certain extent, but erred, in ascribing to the influence derived from the brain and nervous system the vital power of muscular parts. When Hunter's doctrine of the vitality of the blood becomes extended to other fluids of the body, the simplification of disease will be much advanced, which, as our author observes, shall be found to originate in the capillaries, and which, by shewing the intimate connexion between the solids and fluids which constantly are going on in the capillary system, will ultimately annihilate the distinctions of solidists and humoralists.

In his preliminary observations, he says the first step at generalization, which can only be done by induction—

"Is the classification of individual cases of disease into genera and orders, according to the resemblance which their symptoms, observed during life, bear to each other. Those cases which appear the most closely analogous are carefully observed, and their usual history and terminations ascertained, a selection is made from their histories of the symptoms which appear the most characteristic, and are observed the most uniformly to form the definitions (abridged descriptions) of genera of diseases; and from a number of such definitions, a farther selection is made of certain sets of symptoms, which are stated as characterizing a class or order of genera."

Having once settled in our minds clear views of the physiology of all the organs in the living body, and the separate and combined functions of these organs in health, we can account for many of the phenomena of disease, when its history and symptoms are universally admitted; but that this will be limited to a very small number of diseases, appears from what our author adduces.

1st. "The variety of individual cases of disease being, in fact, infinite, and the rules by which they are classed into genera and orders being, for the most part, arbitrary and artificial, it very often happens, that a case which in some of its symptoms, resembles one set of other cases, (and is, therefore, necessarily classed along with them under the same titles) in others of its symptoms bears an equally important resemblance to others, in different parts of the nosology.

There are, therefore, many combinations and successions of symptoms which it is important to study (both for theoretical and practical purposes), besides those on which the arrangements of classes and genera are founded; such are those which we express by the terms tendency to syncope, to coma, or to asphyxia, typhoid tendency, inflammatory tendency, nervous irritability, gastric derangement, putrescent diathesis, &c. which are often observed in the course of many different diseases. When we attempt the explanation of deviation from the healthy state of the body, these morbid states, often common to many cases which are otherwise very dissimilar, claim attention equally as the histories of diseases distinguished by the nosologist, and it becomes obvious that the science of pathology cannot be limited by these arbitrary distinctions.

2dly. When changes in the structure of the body that take place in the course of disease, are examined during life, and more especially after death, it appears that different combinations of symptoms are often apparently excited by the same fundamental diseased action, and consequently found in connexion with the same alteration of organic structure; and again, that very different alterations of structure may be attended in different individuals by symptoms, the greater number of which are very nearly the same."

The great attention which has been bestowed upon morbid anatomy, has led many to consider it as the only source of pathological information, in fact, that pathology and morbid anatomy are synonymous terms. To this opinion they are the more inclined, from the little variety the organic structures exhibit after death, and the precise arrangement which they will admit of. But our author clearly proves that this, though an essential part, constitutes but a small portion of a system of pathology.

1st. "There are very numerous cases, arranged into different genera of disease; some of them important, and even rapidly fatal—*e. g.* different forms of fever, certain cases of apoplexy and syncope, tetanus, &c. &c. which do not uniformly nor necessarily leave behind them, so far as is yet known, any alteration of textures, or other change, perceptible to the anatomist, the pathology of which diseases, therefore, although it may derive assistance from, cannot possibly be founded on the knowledge of morbid appearances.

2dly. In many cases of disease, when decided alterations of structure are found after death, these cannot be connected with the fatal event, and do not furnish a rational explanation of it, without reference to general facts or principles, known to us simply by the previous observation of disease, and by generalization of facts which that study presents. It is only by such observation that we learn, that a certain amount of inflammation over the peritoneum furnishes an adequate explanation of fatal depression of the heart's action; or even, that a certain extent of ulceration of the lungs is sufficient to explain a wasting hectic fever. In such cases it is obvious that the laws, according to which such lesions become injurious or fatal, as they cannot be deduced from the study of the lesions themselves, demand a separate investigation."

In our pathological investigations, our object is, not merely to discover this or that particular hue, which the diseased textures of an organ will exhibit after death, and which will often go far in explaining many of the symptoms which preceded death; but if possible to ascertain the conditions and nature of the diseased action which preceded the structural derangement. Cultivated in this sense only, can we ever expect that pathology will aid us in the treatment of disease.

Our author lays down three general rules which are necessary to the advancement of pathology to a fixed science.

1st. "To have reference to the laws of those morbid actions which produce no lesions of structure.

2nd. Those which precede, and cause such lesions.

3rd. Those which are produced by, and succeed such lesions, or attend their formation."

The author next enters at considerable length into the causes of sudden death, ascribing the fatal effects, either to the "directly depressing or suspending the vital action of the organs of circulation, or else by their obstructing the arterialization of the blood."

The influence which the brain and nervous system exercise over the functions of life, is clearly proved by the fatal effects attending injuries of that system. Thus, syncope or death beginning at the heart, will ensue on a violent shock to the system, which disturbs the nervous powers; coma, or death commencing at the brain, by suspension of sensibility and asphyxia, or death beginning at the lungs, by interrupting the access of air to the blood, for its due arterialization. The experiments of Wilson Philip, and Le Gallois, have shewn that severe injuries to the brain and nervous system will paralyse the actions of the heart and capillaries, and that fatal results unmarked by any appreciable change of structure, will often succeed to severe shocks of the system at large. Thus we see that, though not necessarily essential to the functions of organic life, any violent injury done to the nervous system will certainly disturb or entirely suspend the functions of these organs, but these effects will vary much, according to the constitution of individuals. The author states, from experiments by Chossat, "That the heart's action was for some little time affected by certain injuries of the brain, which checked the circulation in the capillaries so completely as to suspend the secretion and evolution of heat, when the spinal cord, below the neck, in the human body, was severely injured, the circulation of the capillaries has generally appeared for some time, more affected than the heart's action, although it is by gradual failure of circulation that such cases are ultimately fatal."

The author adduces many instances illustrative of these views, but which it is unnecessary here to dwell on more fully; one in particular we shall allude to. A lacerated wound of the abdomen, where, without extensive hæmorrhage, and previous to the appearance of inflammation the patient expires; death in this instance is ascribed by Dupuytren to the exhaustion of sensibility. He next proceeds to notice the effect of heat, lightning, cold, and the action of poisons on the system at large.

"In those," he says, "who become comatose from cold, the heart's action is at the same time enfeebled; and it appears distinctly, from experiments on animals, and observations on the human body, that the most intense cold may be fatal in the same way as a concussion by a direct depressing effect on the circulation; in which case, of course, respiration continues up to the moment when the heart's action ceases; the heart is found motionless, and with arterial blood in its left cavites immediately after death, and the artificial respiration is quite ineffectual in prolonging life.

In all such cases, cold acts as a sedative power on the capillary circulation on the surface, and Dr. Edwards found that its repeated or long-continued application has a peculiar effect in depressing the power of subsequently generating heat. In some instances of frost-bite, the effect is so powerful on the parts to which it is chiefly applied, as to put a final stop to all vital action in them, even where the system at large does not materially suffer; and in many cases, the sedative effect on the vital actions of frost-bitten parts is such, that the inflammation (which is always excited in a greater or less degree by the return of heat to such limbs) shews evident marks of deficient reaction, and tends rapidly to gangrene.

But such effects of intense cold on the vital actions of *individual parts* must be carefully distinguished from the case of cold acting as a powerful sedative on the *whole system;* because in the first of these cases, when the general circulation is strong the chief danger is from the inflammation which is the direct consequence of the restoration of the circulation and natural heat of the parts; and this is chiefly to be moderated by causing that restoration to take place very gradually, therefore chiefly by cold applications tending to retard it; whereas, when the vital power of the whole system has been depressed, there is no such risk of local injury from the restoration of temperature, and external heat and other stimuli may be much more fully applied."

The action of cold on the living system may be viewed under different heads. The first effect arising from its application is that of sensation, which will always be in proportion to the existing state of the sensibility, producing upon the nervous system a shock, according as the impression is quick or slow. Another effect of its application is, the abstraction of heat, which when heat abounds in the animal economy will have a beneficial effect in restoring an equilibrium of temperature, but which must have an injurious effect when the caloric does not rise above the natural standard. It acts on the living fibre as a stimulus, in the ratio of its dryness, and the shortness of its application. Its influence is not confined to the parts to which it is directly applied, but extends to every part of the system, exciting without augmenting the irritability and organic contractility. A few drops of water sprinkled over the face are sufficient to rouse the hysteric patient to a sense of consciousness, and notwithstanding the injurious effects of cold when applied in a low degree of temperature to the living body, we find that its application at 32° Fah. is sufficient to rouse to life hybernating animals.

Another effect of the application of cold is constriction, but which, if long continued, has a contrary effect. Spasm is another result depending upon the disagreeable impression made on the nerves by cold. Such is the effect of its sudden application in some individuals, that Tissot relates a case where a large portion of the intestine was forced out at the anus by spasmodic action. Zimmerman, Hoffman, Pinel, adduce many cases in support of this view. Pinel states that vomiting occurred to a woman immediately on her putting her feet into cold water. Alexander, when he plunged into the river Cydnus, affords us another proof of the spasmodic constriction arising from cold. Upon the reaction which succeeds in vigorous constitutions to the moderate application of cold, we shall not allude, but pass to its benumbing effects, which is the result of severe cold long continued. In this way certain animals have been lulled to sleep, by being kept for some time under the influence of a very low temperature. The blood which leaves the capillaries in this particular state of the system, is driven in greater quantity upon the three great cavities; which, by distending their several vessels oppresses them, disturbs the equilibrium of circulation between the internal and external parts, and excites plethoric engorgements, and many vascular and nervous phenomena, which sometimes terminate fatally. In this way many lunatics have perished, who under the old system of treatment were kept long immersed in cold water, which, by detaining the blood in the head was commonly fatal. The circulation in the lungs and nervous system is so much embarrassed by its influence, that sleep, too often the sleep of death, ensues, whilst, like opium, it destroys all sensibility and contractility. Even the heart under its influence is incapable of further action, having lost all irritability. Prosper Alpinus states, that cold in its results is similar to narcotics. Though this is the general result of cold long applied, there is in Boerhaave's Commentaries, a case of a man who died of cold, whose brain upon dissection shewed evident traces of inflammation in the right hemisphere which was adhering to the dura mater by false membrane. But the physical man is not the only part which suffers from the prolonged application of cold. The intellect, the imagination, the sensitive faculty, become more concentrated, and as it has been said, the soul acts more on herself. To sum up the ordinary impressions of cold,—the circulation becomes languid, the blood passes through the lungs in smaller quantity, less is oxygenated, the pulsations of the heart are in accordance with the small quantity of blood which reaches it, the body grows cold, the blood coagulates, and on dissection, we find it collected in the sinus of the dura mater, the heart, and large vessels.

With respect to the various ways in which poisons exert their fatal influence upon the system, he enters at considerable length. He doubts the conclusions at which many have arrived with respect to their modus operandi—namely, that the sole direct action of poisons is on the nerves of the vessels, and their action on the brain and heart is only sympathetic. He is inclined to the belief, that the effect of those which have been absorbed into the blood, is consequent on their direct application to the more important vital organs. However, he admits that poisons exert some power through the medium of the brain and nerves, more especially when they have been absorbed into the circulation. He rejects altogether the idea of classing poisons according to the mode in which death ensues, which will in general vary with the dose. Oxalic acid acts upon the nervous system, when exhibited in small doses, whilst in large doses, it suspends directly the action of the heart. Arsenic, when taken in large doses, occasions death by its effects upon the heart, before any inflammation ensues, which is the point to be dreaded, when it is exhibited in small doses.

He next proceeds to notice the effects of dangerous hæmorrhage upon the living body, and offers some judicious remarks upon the rapid sinking of the heart's action in violent hæmorrhage. The appearances which the mucous membranes present after death by fasting are ascribed by him to a deficiency of natural intestinal mucus. Upon this point we are not quite satisfied with our author's reasoning, particularly when we consider the different degrees of febrile action which may be excited by the act of fasting, negative as it may appear; neither does it appear to us that he has clearly distinguished cause from effect. If the mucus of the intestines be intended to protect them from the contact of foreign bodies, when taken in under the form of food, when they cease to be taken, the necessity, we should suppose, would no longer exist. But it will be urged, that even still there is a secretion of excrementitious matter, and that then mucus is required; that this may be the case we cannot deny, but we can hardly suppose that Nature, who provides so well in other respects for our existence, should have neglected a provision of this kind. Still, though not satisfied with our author's explanation, we candidly confess that we cannot offer any altogether unobjectionable.

We now come to the consideration of the remote causes of disease. Under this he "includes not only causes acting externally to the body, but also circumstances in the condition of the body itself, previous to the attack of disease in question, which are believed to assist in exciting it." These causes are generally divided into predisponent and exciting. The predisponent are nothing but the tendency which some people have to take on this or that particular form of disease, the exciting is that which calls this disposition into action. Though the same exciting cause will not always produce the same disease, yet the frequency of its occurrence is sufficient to establish it as a general rule.

There is one particular cause, to which the author refers as a fruitful source of disease, and which has often escaped the notice of those who seek in morbid anatomy the cause of all disease, and which, as generally abounding in structural lesions, has been set down as the true cause of disease. Referring to the doctrine of Parry, he observes that the most efficient cause of all diseases is local or general plethora. To follow him through the various causes upon which plethora, general or local, may depend, would, in fact, be but a rescript of the book, to which we would gladly direct the attention of our junior brethren. Neither shall we dwell on the views which he offers, of the operation of contagious and malarious diseases, which, though presenting little that has not been already known, still is deserving attentive perusal. Alluding to the laws which regulate their operation, he says—

"The well-ascertained fact, that the morbific effect of these poisons is remarkably increased by debility and inanition, and diminished by fulness and excitement of the vascular system, gives good reason to believe that their action is consequent on their absorption into the blood; because it appears, that by these circumstances absorption is remarkably increased or diminished."

He next proceeds to consider the deranged action of the heart, which he ranks under the three heads of deficient action, inordinate or irregular action, and painful action. To point out here the common symptoms which attend its deficient action, when syncope ensues, is, we apprehend, unnecessary, being such as all medical men are acquainted with. But there are particular changes which occur in the nervous system not so well understood, and which appear to be chiefly influenced by the condition of the vascular system. This state depends, he says, upon the suddenness with which the depression in the heart's action is effected.

"When it takes place *slowly*, the impulse of the blood on the brain and nerves being very gradually diminished, the nervous system suffers in the first instance very little, and the pulse may become imperceptible, and the skin quite cold, before the senses are obscured, or the intellect sensibly impaired. The senses of sight and hearing are generally the first that are blunted in such cases; but the mind is clear, voluntary motions, though enfeebled, may often be performed with precision;—and the sensation which prompts to acts of respiration is so entire, that a heavy and laborious breathing is gradually produced, evidently depending, not on any impediment to the access of air to the lungs, but simply on the increasing difficulty with which the enfeebled heart propels the blood through the lungs. This is the state of the symptoms in many cases of disease when there is imminent danger of death by syncope. In such cases, the pulsations of the heart become usually more frequent as they become feebler.

But when the heart's action is *rapidly* depressed, as by hæmorrhage, or violent mental emotion, the sudden diminution of the pressure on the brain and nerves, like other sudden changes in the condition of these parts, powerfully affects the functions of the nervous system; and there is vertigo, tinnitus aurium, and the other symptoms attending syncope. In such cases the diminution of sensation is often such that the act of respiration, as well as all perceptible action of the heart, may be nearly or certainly suspended for a time, without any bad consequence.

We therefore see fits of syncope, or a tendency to it, brought on either by loss of blood, or purging, or sweating, or by alteration in the distribution of the blood, as by drawing off the fluid in ascites, we may reasonably infer, that the immediate cause of complete failure of the heart's action is not the mere diminution of the stimulus acting on the heart, but a change in the condition of the nervous system; just as it certainly is when syncope is produced by strong mental emotions, by certain long-continued and unpleasant sensation, such as particular odours, or by intense pain, or the sudden transition from pain to ease, which are likewise frequent exciting causes of this affection."

However intimate this relation, it does not necessarily imply a dependence of the heart's action on nervous influence, though many of the older writers argued strongly in favour of it. That the brain and nervous system extend their influence to the heart is admitted, but not to the extent of regulating its healthy functional movements. Any violent impressions made upon this system will, through its ganglionic connexions, reach the heart, disturbing rather than regulating its movements. In all cases of inordinate action, where organic disease does not exist, or where the muscular substance of the heart is not increased, the indications of cure are, to invigorate the vascular system, in which the nervous generally shares. It is well known that, as the heart becomes weaker, it is more easily excited to contractions, and its functions are sooner disturbed. Under this painful action of the heart, angina is ranked, which, he says, depends immediately on impressions made on the sensitive nerves of the heart. Though not necessarily implying organic disease of the heart, it rarely occurs where such derangement does not exist. But the mode of cure would lead us to suppose that it depended much upon plethora, as we find it always much relieved by local or general bleeding. In irritable weak constitutions, palpitations will often occur, upon a transition from exercise to repose; by which means a larger supply of blood is thrown upon the heart, when the capillary circulation is checked. It is needless to say, that where any mechanical opposition to the heart's action exists, it will be more easily disturbed; when ossification of any of the valves occur, syncope will more frequently happen, which in this condition is pregnant with danger. By means of the stethoscope we can easily ascertain, at a very early period, when disease of this kind exists. In the first place, the heart becomes enlarged, in which the aorta often participates. The state of hypertrophy is proved by the increased impulse which it communicates to the parietes of the thorax. We detect an alteration in the sounds of the heart's action, occasioned by the resistance offered to the current of blood, and the strong efforts of the heart to overcome it.

Our author next enters at considerable length on local determinations and congestions of blood, and their immediate effects.

"They often lead to, or are combined with, not only great increase of exhalations, or of secretions, but likewise with increase of nutrition of healthy textures, or with such provisions of nutrition as constitute morbid or adventitious structures, and we shall find that they often pass into true inflammation, acute or chronic; but without altering the chemical phenomena, or the products from the blood in any part of the body, they may be injurious, or even dangerous in two ways, *first* by disturbance of the functions of the parts when they take place, independently of any lesion of their structure, and consequent derangement of functions of parts, as result from the simple effusion of blood into them."

The doctrine of congestion is not a new one, and though our author has laboured much to shew its effects on the different organs and textures, shewing that there is scarcely a disease to which we are liable, which is not more or less connected with congestion, or at least increased afflux of blood to some particular texture, yet he has added little to what Parry has left us on this subject. Congestion or afflux of blood to any texture or organ does not necessarily imply disease. We know that a larger quantity of blood than ordinarily will often circulate through various textures, and even stagnate for a time in them, without producing disease, such is the distensibility of some textures. It is evident that perfect healthy action is compatible with this congestion; nay, it is a part of the provision of Nature to establish these temporary stagnations of blood, some for nutrition, some for sensations, and others for emotions. To these congestions the mucous membrane, the parenchymatous viscera, the lungs, the liver, spleen, kidneys, &c. are very liable; and even in the brain, notwithstanding what has been urged to the contrary, congestions of blood will sometimes occur and prove fatal. These congestions will of course vary with the age of the individual; in youth we find the capillary system fullest; in advanced life, when the growth of the body is established, the current of blood is directed from the capillary system to internal organs, where congestion will take place when there is any impediment to the free arterialization of the blood; or in the abdominal viscera, when the flow of venous blood is disturbed either in the liver or in the lungs or heart. "Such congestions and effusions are of course most apt to take place in cases where the blood is in full quantity, and has less tendency to coagulation than usual, and therefore such fallacious appearances are seen most frequently after different kinds of violent death, or after contagious febrile diseases of short duration. But when the same appearances are found in parts of the body where gravitation would not determine them, when they are found under the circumstances stated above, as favouring local congestions of blood, and when they correspond to symptoms observed during life, of embarrassment of functions of the parts where they are formed, there can be no difficulty about regarding them as the effects and indications of morbid determinations of blood."

With this brief notice of the important subjects which we have now glanced over, we close our observations for the present number, reserving for the next the subjects of inflammation, idiopathic fever, diseased states of nutrition, diseased states of the nervous system and blood. The style of the author, though not decidedly objectionable, still is evidently laboured, and in many places not very intelligible, from the confusion of parenthesis, a practice which cannot be too strongly condemned. We do not hesitate to state, that were the author as conversant with writing as he is with every department of medical science, the book would be, like his oral lectures, clear, intelligible, and precise.

